# Ocular trauma or Oedipism: completing the evisceration

**DOI:** 10.3205/oc000186

**Published:** 2021-08-04

**Authors:** Ujjwala Narang, Laura Maubon, Vishal Shah, Vijay Wagh

**Affiliations:** 1 St. George’s, University of London, United Kingdom; 2 Moorfields Eye Hospital, London, United Kingdom; 3 King’s College Hospital, London, United Kingdom; 4 St. Thomas’ Hospital, London, United Kingdom

**Keywords:** eye injury, self-mutilation, eye haemorrhage

## Abstract

Self-enucleation, also known as Oedipism, is a rare form of ocular trauma. The objective of this clinical case report is to highlight the importance of diagnosing this unusual injury and thus ensuring its appropriate management. We herein describe a case of a 58-year-old man who presented to hospital with a bleeding left eye. Initially, limited history was provided, however, on further enquiry it was revealed that he had paranoid schizophrenia. The patient eventually had to be sectioned with assistance from the Police and Psychiatry team, upon discovery of self-inflicted mechanism of injury. Subsequently, the patient had evisceration of the left eye and afterwards, demonstrated insight into the pre-operative problems. Literature review emphasizes the importance of close cooperation required between medical specialities to ensure that the underlying cause, usually schizophrenia, is managed in conjunction to the eye injury in order to provide optimum care for the patient.

## Introduction

Ocular trauma is a significant, yet preventable, cause of visual impairment. Annually, there are 55 million eye injuries worldwide [1]; however, it is estimated that 90% of these can be avoided [2]. The mechanism of injury can vary, and a small proportion of these are self-inflicted. These account for approximately 500 cases annually [3]. From a public health perspective, injury to the eye can have devastating consequences, leading to permanent disability and thus result in loss of working capacity. The incidence of blindness due to ocular trauma is approximately 1/100,000 persons/year [1].

Several cases of self-enucleation (self-inflicted removal of the eye) have been described in the literature since the first report in 1846, and have been termed Oedipism [4]. This term has been coined from the Greek tragedy of Oedipus Rex: the character of Oedipus gouges his eyes out upon discovering he has unwittingly killed his father and married his mother. We present an unusual case of ocular trauma with uncertain mechanism on a background of complex mental health and social issues.

## Case description

A 58-year-old man with a bleeding left eye was brought by paramedics, redirected from Accident & Emergency to Eye Casualty in summer 2020 during the COVID-19 pandemic.

Limited history was provided by the patient or known by the paramedic team. The patient was shirtless at presentation and complained of pain. He did not disclose much, except prior assault two years ago which had left the eye with poor sight. He was actively bleeding, and the ocular contents were visibly auto-eviscerating. A collateral history was sought, further examination attempted, and steps were initiated to get the patient prepared for emergency operating theatre.

The collateral history corroborated his report of previous assault, with a history of blunt trauma to the left eye, two years prior, resulting in lens dislocation, anatomical dysregulation, vitreous prolapse and subsequent traumatic uveitis which had left him with perception of light only. Fortunately, the right eye had excellent baseline visual acuity achieving 6/6. Follow-up appointments were not attended, and the patient was lost in the system. It was noted that the patient had a history of paranoid schizophrenia and substance misuse involving methadone. He was currently living in sheltered accommodation and we were advised that he had good engagement at this time with his support services.

**Figure 1:**
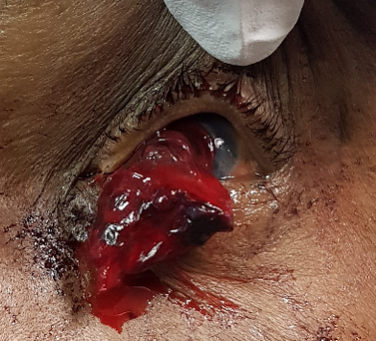
Haemorrhage extending from the cornea of the left eye

On examination, there was an extrusion of actively bleeding ocular contents through a 3 mm central circular corneal perforation (Figure 1 (Fig. 1)) of the left eye. It was not possible to further examine the patient at the time because he was in considerable pain, despite appropriate analgesia. Following multi-level discussion involving the patient, a decision to proceed for an examination under anaesthesia and to stem the bleeding was agreed. The differential diagnoses at this stage included perforation of a corneal ulcer, trauma, and ocular malignancy.

At the time of the listing, the patient was euthymic in mood and demonstrated good understanding of the care plan, he was deemed to have capacity to proceed. A consent form 1 was prepared. As the patient awaited surgery, he had a very sudden change in behaviour. The patient became paranoid, anxious, and no longer cooperative with staff. Despite efforts to rationalise with him, he swiftly left the hospital site, attempts to restrain were not considered and felt inappropriate and unsafe. The police were contacted to locate the patient. Contact was confirmed by the hostel liaison officer when he arrived home early the next day; fortunately his eye had temporarily stopped bleeding. Questions were raised as to the possible mechanism of injury and to the condition of his mental health. Attempts to re-engage the patient to return for further assessment were attempted without success. Furthermore, COVID-19 restrictions made this process of engagement more challenging.

The police decided against placing the patient under Section 136 as he was not deemed to a be an acute threat to himself or others. Two days later, he presented to another ophthalmic institution with further bleeding but again absconded prior to intervention. On the third day following his initial presentation, the hostel staff raised concerns that the injury may have been self-inflicted. CCTV footage from the hostel was reported to show the patient attempting to self-mutilate the left eye with a pair of scissors. At this stage, the police brought the patient to hospital for a mental health assessment and he was sectioned on arrival with support from the Psychiatry team.

The eye was not actively bleeding at this stage, and conservative management with analgesia and antibiotics was initiated. He was also provided psychiatric nursing support during the admission and a detoxing regime. CT imaging excluded any foreign material in his globe. The case was escalated and discussed at a consultant Multi-disciplinary Meeting (MDT). Collectively, it was decided to proceed with left eye evisceration and primary closure without a ball implant to minimise possible further self-mutilation attempts. Pain was identified as a likely motivator and source of distress if the left eye remained untreated. Furthermore, the additional theoretical risk of developing sympathetic ophthalmia, which could risk losing vision in his only seeing eye, was a factor to support evisceration.

With the support of the psychiatric team, the patient became more communicative and was able to retain capacity and consent for the surgery as initially planned. Surgery was successful and routine. It was immediately evident during post-operative bedside review that the patient’s mood had improved considerably. He was engaged and pleased to be receiving both ophthalmic and psychiatric support. He demonstrated insight into the pre-operative problems and was grateful for the interventions he had received. The importance of complying with post-operative care was relayed to him and was undertaken with ongoing mental health support services. Post-operatively he was transferred to an in-patient Psychiatry ward.

At 5 months post-op, he is still compliant, and his mental health condition has been well managed.

## Discussion

Self-enucleation, also known as Oedipism, is an uncommon form of ocular self-trauma but is being seen more frequently in the literature, mostly as case reports. In a review of 50 cases of self-enucleation described by Krauss et al. [5], 19 cases were bilateral, and 31 cases were unilateral. The most common method of self-enucleation was digital trauma, occurring in 20 cases, but tools such as scissors and screwdrivers have previously been documented. Our patient did claim to rub his eye very hard due to irritation and knowledge of previous dysregulated anatomy may have made perforation much easier. There is no definitive gender predilection and is most common in persons aged 40–50 years. Most of the patients described by Krauss et al. [5] had a psychiatric diagnosis, as 50% had chronic depression and 50% also had schizophrenia. Unlike our patient who was relatively euthymic in presentation, many major self mutilations may be associated with religious and/or sexual delusions [6]. Substance misuse is also common in the history [6].

In our differential we considered corneal perforation secondary to infective ulcer, but we did not find evidence of this during surgery. Neither were there any features of ocular malignancy.

The history in the case was vague and challenging. It is important to have a low threshold for suspicion when the history does not seem plausible. However, it is also important to respect patients who give vague histories as they may have many reasons to not being forthcoming. Factors to withhold information can be as simplistic as anxiety, embarrassment or more complex involving possible domestic abuse, legal, financial implications and mental health or memory concerns. Our patient had mental health factors which may have spiralled downhill. It is not clear if his pain could have been a key factor for his deteriorating health or whether a combination of substance misuse, the current pandemic and general mental demise was the primary driver. Moreover, it is worth noting that the complexities of mental capacity assessment are best undertaken with the support of psychiatric services. On this occasion, despite active mental health problems, he was able to maintain his capacity to consent, which was an important nuance in this case. Unfortunately, patients do not present as they do in textbooks, and history taking is an art. Treating a whole person is a lesson we are often taught at medical school and in this case, we were able to achieve this.

## Conclusion

Self-inflicted ocular trauma is, thankfully, a relatively rare entity. This case highlights the importance of close cooperation required between different medical specialties to ensure optimum care of an often severely disturbed patient. Psychiatrists can play a significant role in addressing affective, behavioural, and cognitive triggers of ocular self-injury, which can help guide ophthalmologists. In conclusion, the prevention of self-enucleation depends on the identification and timely management of the underlying cause, usually a psychiatric condition, and close observation to prevent future self-harm.

## Notes

### Acknowledgements

The authors would like to thank Jack Gormley, Joshua Harvey, Miss Ailsa Ritchie, Miss Susie Morley, Mr. Danny Morrison, Prof. Tom Williamson, Prof. Miles Stanford, the psychiatry team, and the medical team.

### Informed consent

Informed consent has been obtained from the patient for the publication of this case report.

### Competing interests

The authors declare that they have no competing interests.
